# Enlarged Tonsils

**Published:** 1903-10-10

**Authors:** 


### Hospital Clinics and Medical Progress.

### ENLARGED TONSILS.

Mr. Lawrie H. McGavin[1] puts in a strong plea for the employment of more conservative methods than are now commonly used in dealing with enlarged tonsils. It is too much the rule, he believes, to look upon removal as the only really efficient treatment in these cases, whereas many are quite amenable to milder measures. A proper recognition of this fact is most desirable. As he very rightly points out, although we do not know exactly of what use the normal tonsil may be in the animal economy, and in spite of the fact that individuals seem to get on all right after both their tonsils have been removed, yet it is not right to assume on these grounds alone that the tonsils are functionless organs and serve no useful purpose whatever. Mr. McGavin is of opinion that there is a good deal of evidence to show that the tonsils ought to be looked upon as scavengers of the oro-pharynx, and mentions the following points in support of that theory:—(1) They are situated at the back of the mouth just where the movements of the tongue are least efficacious in cleansing the buccal cavity. (2) The bolus of food in its passage to the pharynx is compressed between the faucial and lingual tonsils and is thus probably denuded to a great extent of surface bacteria, the compression of the tonsil at the same time possibly serving to cause the exudation of a protective film of lymph by which any organisms may be rendered innocuous during the subsequent passage over the posterior surface of the arytænoid cartilages. (3) The fact that the tonsil is a lymphatic gland placed in a septic cavity, and that it is in direct communication with that cavity, suggests that its duty is that of rapid phagocytosis without the mediation of lymphatic vessels. (4) The presence of so much lymphatic tissue, of which the faucial tonsils form only a portion, in the oro-pharynx is evidence of the necessity for protection against bacterial invasion in this situation. Whether the tonsils are important functional organs or not, Mr. McGavin is most likely correct when he says that removal is often performed in cases which might quite satisfactorily be treated by properly applied palliative measures, and that frequently both tonsils are submitted to the guillotine when it would suffice to remove only one. Of the many causes of tonsillar enlargement he draws attention to the following as being those in which mistakes are commonly made, and in which it is desirable to indicate some definite line of treatment.

1. *Simple Hypertrophy of the Tonsil.*—Children who suffer from this not uncommon condition are usually brought by their parents on account of snoring. On examination the tonsils are found to be large, smooth, normal in colour, with no distension of the crypts, no exudation of lacunar secretion, and no rugosity pointing to the presence of chronic fibroid change. Such tonsils cannot be satisfactorily treated except by removal, and this is indicated both on account of the partial obstruction to respiration and the danger of suffocation which may occur if an acute attack of tonsillitis ensues.

2. *Enlargement of the Tonsil due to continued Irritation.*—The commonest cause of this condition is naso-pharyngeal obstruction, and the consequent passage of bacteria and dust-laden air through the oro-pharynx instead of through the nose. In these cases, provided that they are not of sufficiently long standing for the tonsils to have become hard and fibrous from continued irritation, it is only necessary to remove the obstruction to nasal respiration and the tonsils will probably resume their normal condition. But if they have already become hard and fibrous, ablation will be necessary. The latter operation is also indicated if there is obvious enlargement of the cervical lymphatic glands.

3. *Enlargement accompanied by Lacunar Inflammation.*—This condition is most commonly seen in young adults, and is one of great discomfort on account of the frequent spitting up of foul epithelial plugs and the bad odour which is imparted to the breath. Conservative treatment is indicated in these cases if the diseased crypts are neither numerous or large, if the upper part of the tonsil is healthy and the supratonsillar fossa, the importance of which has been pointed out by Watson Williams, is shallow, and if the disease has not been of long standing. The method which Mr. McGavin adopts in cases which fulfil the above conditions is as follows:—A 5 per cent. solution of cocaine is applied to the tonsil, and with a small scoop the contents of the crypt are cleared out. The cavity is then cleaned with a small pledget of cotton-wool, anæsthetised with a 20 per cent. solution of cocaine, and thoroughly cauterised with an electric cautery point at a dull red heat. Not more than two or three crypts should be treated in this manner at one sitting or the subsequent inflammation may be excessive. The results of this treatment he has found to be perfectly satisfactory.

[1] Lancet, Sept. 26.

